# Mortality in Young Adults following *in Utero* and Childhood Exposure to Arsenic in Drinking Water

**DOI:** 10.1289/ehp.1104867

**Published:** 2012-09-04

**Authors:** Allan H. Smith, Guillermo Marshall, Jane Liaw, Yan Yuan, Catterina Ferreccio, Craig Steinmaus

**Affiliations:** 1 Arsenic Health Effects Research Program, School of Public Health, University of California, Berkeley, Berkeley, California, USA; 2 Departamento de Salud Pública, Escuela de Medicina, and; 3 Departamento de Estadística, Facultad de Matemáticas, Universidad Católica de Chile, Santiago, Chile; 4 Office of Environmental Health Hazard Assessment, California Environmental Protection Agency, Oakland, California, USA

**Keywords:** arsenic, childhood exposure, Chile, drinking water, environmental exposure, *in utero*, mortality

## Abstract

Background: Beginning in 1958, the city of Antofagasta in northern Chile was exposed to high arsenic concentrations (870 µg/L) when it switched water sources. The exposure abruptly stopped in 1970 when an arsenic-removal plant commenced operations. A unique exposure scenario like this—with an abrupt start, clear end, and large population (125,000 in 1970), all with essentially the same exposure—is rare in environmental epidemiology. Evidence of increased mortality from lung cancer, bronchiectasis, myocardial infarction, and kidney cancer has been reported among young adults who were *in utero* or children during the high-exposure period.

Objective: We investigated other causes of mortality in Antofagasta among 30- to 49-year-old adults who were *in utero* or ≤ 18 years of age during the high-exposure period.

Methods: We compared mortality data between Antofagasta and the rest of Chile for people 30–49 years of age during 1989–2000. We estimated expected deaths from mortality rates in all of Chile, excluding Region II where Antofagasta is located, and calculated standardized mortality ratios (SMRs).

Results: We found evidence of increased mortality from bladder cancer [SMR = 18.1; 95% confidence interval (CI): 11.3, 27.4], laryngeal cancer (SMR = 8.1; 95% CI: 3.5, 16.0), liver cancer (SMR = 2.5; 95% CI: 1.6, 3.7), and chronic renal disease (SMR = 2.0; 95% CI: 1.5, 2.8).

Conclusions: Taking together our findings in the present study and previous evidence of increased mortality from other causes of death, we conclude that arsenic in Antofagasta drinking water has resulted in the greatest increases in mortality in adults < 50 years of age ever associated with early-life environmental exposure.

Millions of people worldwide are exposed to arsenic in their drinking water, and arsenic is a well-documented cause of many serious health effects. The International Agency for Research on Cancer (2004) classified arsenic in drinking water as carcinogenic to humans, based on evidence that arsenic causes cancers of the skin, lung, and bladder. Chronic arsenic exposure has also been shown to cause noncancer health outcomes in multiple organs, including reproductive, cardiovascular, pulmonary, neurologic, and dermal effects (National Research Council 1999, 2001). In the present study we investigated all causes of death following probable *in utero* and early-life exposure to arsenic.

We examined mortality in an area in northern Chile that has some unique features that make it an ideal location to study long-term outcomes from arsenic exposure. It is the driest inhabited place on earth (McKay et al. 2003). Because it had no private wells, all residents drank water from the only available source: the city water supply. Antofagasta obtained drinking water from rivers that flow from springs in the Andes Mountains. Before 1958, the arsenic level of the city water supply was about 90 µg/L. In 1958, a new city water supply was installed using water from the Toconce and Holajar rivers, which contained 800 and 1,300 µg/L of arsenic, respectively (Smith et al. 1998). With these new sources of water, the average arsenic concentration in the city water supply increased dramatically to 870 µg/L. After a water treatment plant was installed in 1970, the arsenic concentration in the city water supply dropped to 110 µg/L for about 10 years and was reduced further thereafter. The water supply now contains < 10 µg/L of arsenic.

In previous studies of mortality among adults 30–49 years of age, we discovered increased mortality due to lung cancer and bronchiectasis (Smith et al. 2006), kidney cancer (Yuan et al. 2010), and acute myocardial infarction (Yuan et al. 2007) among residents born during or shortly before the high-exposure period. We therefore decided to extend our analysis to assess mortality from all causes of death among adults 30–49 years of age who were born during or before the high-exposure period. Because the age range of 30–49 years is a young age at which to die, we refer to these deaths as deaths in “young adults.”

## Methods

We previously reported the methods used for mortality studies in northern Chile (Smith et al. 2006; Yuan et al. 2010). In brief, we obtained computerized mortality data for 1989–2000 from the Ministry of Health (Santiago, Chile) for all 13 regions of Chile. Deaths were divided into two groups: residents of Antofagasta and neighboring Mejillones (cities located in Region II of Chile that have the same water source), and residents in all other regions of Chile. Antofagasta is very much larger than Mejillones, so here we refer to Antofagasta and Mejillones combined as Antofagasta. We selected 1989 as the first year because it is the first year for which deaths in Antofagasta were reported separately from the rest of Region II. Other parts of Region II also had arsenic in drinking water, although to a lesser extent than Antofagasta. Outside of Region II, the rest of Chile has not been exposed to high levels of arsenic in drinking water. With rare exceptions, arsenic concentrations in water sources outside of Region II have been < 10 µg/L. In a 1984 nationwide survey of 2,000 people, average urine arsenic concentrations were 14 µg/L (Venturino 1991). This is similar to average levels found in the general U.S. population (16.7 µg/L) and indicates very low arsenic concentrations in drinking water (Steinmaus et al. 2009). In addition to mortality data, we obtained survey and census data comparing variables such as smoking, diet, and socioeconomic status from Antofagasta and the rest of Chile in order to evaluate potential confounding from these factors.

In the present study, we focused on deaths during 1989–2000 among people 30–49 years of age from Antofagasta or from all of Chile except Region II (referents). People from Antofagasta in this age range would have been *in utero*, children, or adolescents (up to 18 years of age) during at least part of the high-exposure period of 1958–1970. Two birth cohorts were defined for this investigation: births during 1958–1970 (probable *in utero* and childhood exposure to those born in Antofagasta), and births during 1940–1957 (probable childhood, but not *in utero*, exposure to those born in Antofagasta).

The Ministry of Health coded causes of death for 1989–1998 using the *International Classification of Diseases, 9th Revision* (ICD-9; World Health Organization 1978) and for 1999–2000 using the 10th revision (ICD-10; World Health Organization 1992). For our analysis, we converted all ICD-10 codes into ICD-9 codes. In our initial analysis, we noticed that several causes of death listed under ICD-9 codes 580–589 (genitourinary) exhibited elevated standardized mortality ratios (SMRs). The excess deaths were limited to chronic renal failure (ICD-9 code 585), renal failure unspecified (ICD-9 code 586), chronic glomerulonephritis (ICD-9 code 582), and renal sclerosis (ICD-9 code 587). Because each of these causes of death relates to chronic renal disease and renal failure, we grouped them together as mortality from chronic renal disease.

We obtained annual estimates of the population living in Antofagasta in Region II, as well as for the rest of Chile excluding Region II, for 1989–2000 from the National Institute of Statistics (Instituto Nacional de Estadísticas); estimates were stratified by age and sex. We calculated SMRs for deaths among those 30–49 years of age, using 10-year age groups (30–39 and 40–49 years) for age standardization. We calculated significance and confidence intervals (CIs) based on the Poisson distribution (Selvin 1995). In view of the clear direction of the *a priori* hypotheses for arsenic causing increased risks for both malignant and nonmalignant diseases, here we present one-tailed tests of statistical significance. We tested for effect modification by age group (comparing 30- to 39-year and 40- to 49-year age groups) and effect modification by sex using Poisson regression interaction terms with two-tailed tests. We also tested for effect modification by birth period, comparing mortality for those born in 1940–1949 with those born in 1950–1957, but because there were no significant differences by birth period for any outcome (*p* > 0.05), we report results for both periods combined. We compared mortality at 30–49 years of age among those born in 1940–1957, who would have experienced at least part of their exposure ≤ 18 years of age, with mortality among those born during 1958–1970, most of whom would have been exposed *in utero* if they were born in Antofagasta.

## Results

Based on a 1990 random sample survey of Chilean cities that included Antofagasta [Caracterizacion Socio Economica Nacional; (CASEN) 1990], the prevalence of smoking in 1990 was comparable between Antofagasta and the rest of Chile (Table 1). Demographic characteristics for Region II from the 2002 Census (Instituto Nacional de Estadisticas Chile 2002) are similar for Region II and Chile as a whole, including the percentage of the population living in urban areas (98% and 87%, respectively) and the percentage of medically certified death certificates (90% and 86%). Diet and other health risk factors collected in studies of stratified random population census samples conducted in 2003 and 2009 (Gobierno de Chile, Ministerio de Salud 2003, 2010), including obesity, blood cholesterol, glucose, and hypertension, were also similar between the comparison populations.

**Table 1:** Comparing smoking data, demographic variables, and risk factors for Region II (of which Antofagasta constitutes more than half of the total population) with those for all of Chile.

Variable	Region II	All of Chile
Smoking (%)a
Nonsmokers	78.0	77.5
Moderate smokers (> 0 to 1 pack/day)	21.0	21.1
Heavy smokers (> 1 pack/day)	1.0	1.2
Men smokers	27.4	26.6
Women smokers	16.6	19.3
Demographic variable (%)b
Urban	98	87
Catholic	72	70
Literate	98	97
Prebasic education	4	4
University/professional education	17	14
Death certificate certified by physician	90	86
Medical risk factorc
Average BMI (kg/cm2)	27.6	26.8
Obese [BMI > 30 (%)]	19.2	21.9
Morbidly obese [BMI > 40 (%)]	2.8	1.3
Average HDL cholesterol (mg/dL)	34.2	44.6
Average LDL cholesterol (mg/dL)	105.0	115.4
Hypertension [blood pressure > 140/90 (%)]	28.9	33.5
Average total cholesterol (mg/dL)	174.0	186.0
Average serum glucose (mg/dL)	85.8	92.9
Diabetes (%)	3.2	4.2
Regular exercise (%)	13.8	9.2
Dietary risk factor in national surveyd
Alcohol consumed per day (g)	41.5	55.6
Fruit/vegetables consumed per day (g)	174.0	186.0
Salt consumption/day (g)	173.8	185.8
Abbreviations: BMI, body mass index; HDL, high-density lipoprotein; LDL, low-density lipoprotein. aData from CASEN (1990). bData from Instituto Nacional de Estadisticas Chile (2002). cData from Gobierno de Chile, Ministerio de Salud (2003). dData from Gobierno de Chile, Ministerio de Salud (2010).				

We first estimated SMRs comparing specific causes of death among adults 30–49 years of age born in Antofagasta during 1940–1970 (both sexes combined) to the same age group born in the rest of Chile (data not shown). We observed no increased mortality from infectious and parasitic diseases (ICD-9 codes 001–139; SMR = 1.0; 95% CI: 0.8, 1.3), endocrine and nutritional diseases (ICD-9 codes 240–279; SMR = 1.2; 95% CI: 0.7, 1.8), for diseases of the respiratory system (ICD-9 codes 460–519; SMR = 1.1; 95% CI: 0.9, 1.3), or diseases of the digestive system (ICD-9 codes 520–579; SMR = 0.8; 95% CI: 0.7, 0.9). Mortality was increased for all cancers combined (ICD-9 codes 140–239; SMR = 1.7; 95% CI 1.6, 1.9; *p* < 0.001), deaths from circulatory diseases (ICD-9 codes 390–459; SMR = 1.7; 95% CI: 1.5, 2.0; *p* < 0.001), and diseases of the genitourinary system (ICD-9 codes 580–629; SMR = 2.0; 95% CI: 1.5, 2.8; *p* < 0.001).

Table 2 presents the SMRs for selected causes of death in Antofagasta for males and females 30–49 years of age during 1989–2000 and the expected numbers estimated from those in the rest of Chile (excluding Region II). The “all other cancers” category comprises all cancers other than those of the bladder, larynx, and liver, as well as lung and kidney cancers, which we previously reported to be associated with early-life arsenic exposure (Smith et al. 2006; Yuan et al. 2010). Mortality for all cancers combined was increased for males born during 1940–1957, most of whom would have experienced at least some exposure before 18 years of age (SMR = 2.1; 95% CI: 1.9, 2.4; *p* < 0.001), and for those born during the high-exposure period (1958–1970), most of whom would have experienced both *in utero* and childhood exposure (SMR = 2.2; 95% CI: 1.7, 2.8; *p* < 0.001). Mortality from all cancers combined was also increased among females (SMR = 1.4; 95% CI: 1.2, 1.6 and SMR = 1.4; 95% CI: 1.1, 1.8 for those born in 1940–1957 and 1958–1970, respectively), but to a lesser extent than in males.

**Table 2:** Observed and expected deaths and SMRs for males and females 30–49 years of age during 1989–2000 and born in Antofagasta, Chile, in 1940–1957 and 1958–1970 (during the high-exposure period).

Cancer	Sex	Born 1940–1957								Born 1958–1970								*p* for interaction^b^
		Observed	Expected	SMR (95% CI)		*p*-Value^a^	Observed	Expected	SMR (95% CI)		*p*-Value^a^							
All cancer	Male	226	105.3	2.1	(1.9, 2.4)	< 0.001	69	30.8	2.2	(1.7, 2.8)	< 0.001
Female	219	154.5	1.4	(1.2, 1.6)	< 0.001	59	41.3	1.4	(1.1, 1.8)	< 0.01	0.83
Bladder cancer	Male	11	0.8	13.7	(6.8, 24.5)	< 0.001	6	0.1	65.7	(24.1, 143)	< 0.001
Female	2	0.3	7.9	(1.0, 28.6)	0.03	3	0.1	43.0	(8.9, 126)	< 0.001	0.01
Laryngeal cancer	Male	7	0.8	8.9	(3.6, 18.3)	< 0.001	1	0.0	27.4	(0.7, 153)	0.04
Female	0	0.1	—		—	0	0.0	—		—	0.53
Liver cancer	Male	10	4.1	2.4	(1.2, 4.4)	0.01	5	0.9	5.9	(1.9, 13.7)	< 0.01
Female	6	4.1	1.5	(0.5, 3.2)	0.23	4	0.9	4.7	(1.3, 12.0)	0.01	0.04
All other cancersc	Male	79	82.9	1.0	(0.8, 1.2)	0.64	41	27.7	1.5	(1.1, 2.0)	0.01
	Female	174	142.1	1.2	(1.0, 1.4)	< 0.01	48	39.0	1.2	(0.9, 1.6)	0.09	0.36
Chronic renal diseased	Male	14	7.5	1.9	(1.0, 3.1)	0.02	6	2.7	2.3	(0.8, 4.9)	0.05
Female	14	7.1	2.0	(1.1, 3.3)	0.02	6	2.4	2.5	(0.9, 5.4)	0.04	0.71
All other noncancer deaths minus injuriese	Male	310	367.7	0.8	(0.8, 0.9)	0.99	110	128	0.9	(0.7, 1.0)	0.94
	Female	187	178.3	1.0	(0.9, 1.2)	0.27	89	61.8	1.4	(1.2, 1.8)	< 0.01	0.33
All data presented for Antofagasta include neighboring Mejillones, which had the same water sources. aOne-sided p-value. bTwo-sided p-value; test for interaction between birth periods 1940–1957 and 1958–1970 adjusted for sex. c“All other cancers” comprises all cancers except those of the bladder, larynx, liver, lung, and kidney. dICD‑9 codes 580–589. e“All other noncancer deaths minus injuries” comprises all noncancer deaths except injuries, acute myocardial infarction, bronchiectasis, and other COPD; the last three of these diseases were previously shown to be associated with early-life arsenic exposure (Smith et al. 2006; Yuan et al. 2007).																						

Mortality from bladder cancer was greatly increased in males and females (Table 2), particularly among those born during 1958–1970, the high-exposure period with probable exposure *in utero* (for males, SMR = 65.7; 95% CI: 24.1, 143; for females, SMR = 43.0; 95% CI: 8.9, 126; *p* = 0.01 for interaction between birth periods 1940–1957 and 1958–1970 for males and females combined, adjusted for sex). Increases in liver cancer mortality were also more pronounced for those born during 1958–1970 (for males, SMR = 5.9; 95% CI: 1.9, 13.7; for females, SMR = 4.7; 95% CI: 1.3, 12.0; *p* = 0.04 for interaction by birth period among males and females combined). Mortality from laryngeal cancer was increased among men born in 1940–1957, before the high-exposure period (SMR = 8.9; 95% CI: 3.6, 18.3).

Among noncancer causes of death for adults 30–49 years of age, we observed evidence of increased mortality from chronic renal disease, with SMRs in the range of 1.9–2.5 for men and women born during or before the high-exposure period (Table 2).

**Figure 1:**
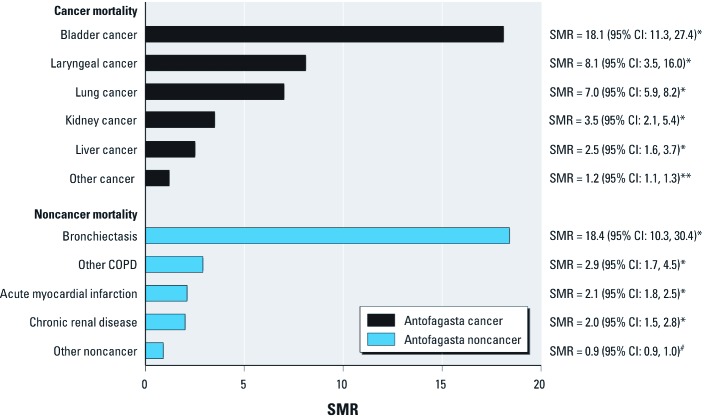
Summary of SMRs for 30–49-year-old males and females (pooled) who were born in Antofagasta, Chile, combining those born before and during the high-exposure period (Smith et al. 2006; Yuan et al. 2007, 2010). *p ≤ 0.001. **p = 0.002. ^#^*p* = 0.93.

When data for both time periods were combined, we observed no significant differences in SMRs by sex for outcomes evaluated for the first time in the present study, or for outcomes evaluated previously using SMRs for different time periods or separately for men and women [lung cancer, bronchiectasis, and other chronic obstructive pulmonary disease (COPD) (Smith et al. 2006); acute myocardial infarction (Yuan et al. 2007); and kidney cancer (Yuan et al. 2010)] (Figure 1). The highest combined SMRs were for bladder cancer (SMR = 18.1; 95% CI: 11.3, 27.4) and bronchiectasis (SMR = 18.4; 95% CI: 10.3, 30.4) (Figure 1).

## Discussion

We found increases in mortality from several different causes of death in the 30- to 49-year-old study population. We previously reported increases in lung cancer and bronchiectasis in Chile following early-life arsenic exposure (Smith et al. 2006), as well as increases in bronchiectasis in India after adult exposure (Guha Mazumder et al. 2005). We have also reported increases in mortality from kidney cancer (Yuan et al. 2010) and myocardial infarction (Yuan et al. 2007) after early-life exposure to arsenic in Antofagasta. Bladder cancer SMRs from the present analysis for adults (30–49 years of age) born during the high-exposure period (1958–1970; males: SMR = 65.7; 95% CI: 24.1, 143, females: SMR = 43.0; 95% CI: 8.9, 126) are 5–10 times higher than those we reported previously for all ages combined, regardless of age at exposure (males, 6.0; females, 8.2) (Smith et al. 1998).

The association between early-life exposure to arsenic and mortality from laryngeal cancer suggests that this cancer might be related to arsenic exposure, although the number of laryngeal cancer deaths was very small and the association was evident only among men.

The findings concerning mortality from chronic renal disease were unexpected. In one study in Taiwan, Chiu and Yang (2005) reported a relationship between arsenic and mortality from renal diseases, but the authors did not specifically investigate early-life exposure. In the study area in Taiwan, residents had consumed arsenic-contaminated well water with a median concentration of 780 µg/L and had renal disease mortality rates that were 50% higher than those in unexposed populations. It is plausible that renal disease might relate to arsenic in water because arsenic is excreted through the kidney and is also a probable cause of kidney cancer (National Research Council 2001; Yuan et al. 2010).

Adult liver cancer has been linked to arsenic exposure, primarily in studies in Taiwan (Chiu et al. 2004). In a previous study in Region II of Chile, we found little evidence of increased mortality from liver cancer in adults (Smith et al. 1998), but we did not evaluate early-life exposure. However, in a subsequent analysis of childhood cancers (Liaw et al. 2008), liver cancer mortality in children 10–19 years of age was increased in Region II compared with Region V [for males, relative risk (RR) = 8.9; 95% CI: 1.7, 45.8; *p* = 0.009; for females, RR = 14.1; 95% CI: 1.6, 126; *p* = 0.018]. The data we present here are, to our knowledge, the first to link early-life arsenic exposure to liver cancer mortality in adults 30–49 years of age.

A major strength of this study is the large size of the exposed population: There were > 125,000 residents in Antofagasta and Mejillones in 1970 who were exposed to water arsenic concentrations of 870 µg/L, including approximately 60,000 children with early-life exposure during the high-exposure period. The largest cohort study on arsenic conducted in Taiwan involved only 698 subjects ≥ 40 years of age exposed to arsenic concentrations > 300 µg/L (Chiou et al. 2001). Recently published cohort studies in Bangladesh involved 10,431 subjects ≥ 15 years of age exposed to arsenic at 300 µg/L, the largest study (Sohel et al. 2009), and 2,889 subjects ≥ 18 years of age exposed to > 150 µg/L, in the second largest study (Argos et al. 2010). In addition, the populations studied in Taiwan, India, and Bangladesh received their water from a large number of small-town or domestic wells with wide variations in arsenic concentrations, even between closely located wells (Guha Mazumder et al. 1998; Van Geen et al. 2002), making it extremely difficult to accurately estimate early-life exposure decades earlier.

One potential weakness of the present study is that it is ecological, comparing Antofagasta with the rest of Chile. However, this study does not have the usual problems associated with what is sometimes termed the “ecologic fallacy” (Morgenstern 1995) because the one source of drinking water in Antofagasta had a known concentration of arsenic; therefore, we can be confident that virtually everyone who lived in Antofagasta during the high-exposure period was indeed exposed. Migration into and out of the study area could also introduce bias, but people migrating from Antofagasta to elsewhere in Chile would constitute a very small proportion of the total Chilean population. Any resulting bias would tend to reduce relative mortality estimates for Antofagasta. Migration into Antofagasta also would tend to bias estimates toward the null because these residents would be misclassified as exposed even though they did not reside in Antofagasta during the high-exposure period. In addition, migration within Chile is relatively uncommon: From 1965 to 2000, annual internal migration among regions of Chile was only 0.6% compared with 1.2% in Argentina, 3.1% in the United Kingdom, and 6.6% in United States (Soto and Torche 2004).

Another potential weakness of the study is the use of death certificate data. Based on Chile’s Census 2002 data (Instituto Nacional de Estadisticas Chile 2002), most death certificates (86%) in the country are signed by physicians; in Region II, where Antofagasta is located, the percentage is similar (90%). In addition, Chile has a national health care system that services the whole country (Reichard 1996). Therefore, it is unlikely that differences in diagnostic practices between Antofagasta and the rest of Chile would produce spurious differences in mortality rates.

The importance of ecological studies in causal inference concerning arsenic in drinking water was recognized by the International Agency for Research on Cancer (2004):

For most other known human carcinogens, the major source of causal evidence arise from case–control and cohort studies, with little, if any, evidence from ecological studies. In contrast, for arsenic in drinking-water, ecological studies provide important information on causal inference, because of large exposure contrasts and limited population migration.

There are two reasons why confounding is not a concern, the first reason of which involves the magnitude of the mortality rate ratios identified. Consider, for example, the SMRs for acute myocardial infarction mortality of 2.3 and 2.7 among Antofagasta men for births during 1940–1957 and 1958–1970, respectively. These values are comparable to the acute myocardial infarction mortality rate ratio of 2.11 for current cigarette smokers who smoked > 20 pack-years compared with never-smokers from a large cohort study of about 140,000 men in the United States (Henderson et al. 2007). These similarities suggest that confounding by smoking would explain our SMRs only if all men in Antofagasta smoked but no men in the rest of Chile smoked. Similarly, confounding by smoking would not explain the estimated SMRs for lung cancer, laryngeal cancer, and bladder cancer.

The second reason confounding is not a major concern is that there is no evidence of major differences in risk factors (other than arsenic) between Antofagasta and the rest of Chile. For example, a survey conducted in 1990 indicated that the prevalence of smoking in Region II (27.4% in men and 16.6% in women) was similar to Chile as a whole (26.6% in men and 19.3% in women) (Marshall et al. 2007). It is also extremely unlikely for other confounding factors, including diet or exercise, to produce the magnitude of the SMRs we report here. As shown in Table 1, other cardiovascular mortality risk factors, including body mass index, obesity, cholesterol, and hypertension, were not substantially different between Region II and all of Chile in 2003 (Gobierno de Chile, Ministerio de Salud 2003). Having given consideration to all potential sources of bias, we conclude that our study provides strong epidemiological evidence of increased mortality risks from several causes in young adults exposed to arsenic in early life.

Inorganic arsenic and its metabolites readily pass through the placenta, exposing the fetus to concentrations similar to those of the mother (Concha et al. 1998). Animal experiments have shown that arsenic is a transplacental carcinogen in mice and causes tumors in offspring (Tokar et al. 2011; Waalkes et al. 2007). Vahter (2008) has shown that arsenic acts epigenetically and interferes with DNA methylation. Ren et al. (2010) reported that arsenic exposure may alter DNA methylation, globally affecting the expression of multiple genes; this may explain why exposure to arsenic is associated with multiple disease outcomes in different organs.

We know of no childhood environmental exposure that results in comparable increases in adult mortality rates (Figure 1). Yorifuji et al. (2010) reported that mortality from pancreatic cancer and leukemia were increased in young adults after arsenic exposure from contaminated milk powder in Japan; these exposures were very high and resulted in acute poisoning effects. In a study of 60,182 people, Vineis et al. (2005) reported elevated lung cancer risks from childhood passive smoking, but the relative risk from “daily, many hours” of passive smoking exposure was 3.63 (95% CI: 1.19, 11.11) and there were only four cases of lung cancer in this group. Other studies of passive childhood smoking have not found increased risks (Boffetta et al. 2000). A nonenvironmental exposure—radiation treatment of childhood cancer—causes major increases in later mortality from other cancers (excluding recurrence of the treated cancer) as well as from noncancer outcomes. A recent report from the Childhood Cancer Survivor Study showed that mortality from other cancers was increased [relative risk (RR) = 2.9; 95% CI: 2.1, 4.2] and that mortality from cardiac causes (RR = 3.3; 95% CI: 2.0, 5.5) and “other” causes (RR = 2.0; 95% CI: 1.3, 3.1) were also increased (Mertens et al. 2008). Increased cancer mortality has also been demonstrated in atomic-bomb survivors exposed *in utero* or as young children (Preston et al. 2008). Excluding these fairly rare and specific high-dose radiation exposure scenarios, our findings suggest that early-life exposure to arsenic in drinking water results in greater increases in mortality in adults < 50 years of age than those attributable to any other early-life environmental exposure.

## Conclusions

To our knowledge, this is the first investigation of all causes of death in young adults following early-life exposure to arsenic in drinking water. In those exposed to water arsenic concentrations of approximately 870 µg/L, we identified pronounced increases in mortality in young adults 30–49 years of age from cancers of the bladder, larynx, and liver and from renal diseases associated with chronic renal failure. Taken together with the increased mortality from other causes (Smith et al. 2006; Yuan et al. 2007, 2010), the magnitude and extent of the increased mortality we have identified are without precedent for any early-life environmental exposure. Our findings need to be confirmed in other populations, but they add strong support for efforts to reduce population exposure to arsenic in drinking water, particularly during pregnancy and childhood.
